# Isolation and complete genome analysis of neurotropic dengue virus serotype 3 from the cerebrospinal fluid of an encephalitis patient

**DOI:** 10.1371/journal.pntd.0006198

**Published:** 2018-01-12

**Authors:** Rama Dhenni, Mulya Rahma Karyanti, Nina Dwi Putri, Benediktus Yohan, Frilasita A. Yudhaputri, Chairin Nisa Ma'roef, Araniy Fadhilah, Aditya Perkasa, Restuadi Restuadi, Hidayat Trimarsanto, Irawan Mangunatmadja, Jeremy P. Ledermann, Ronald Rosenberg, Ann M. Powers, Khin Saw Aye Myint, R. Tedjo Sasmono

**Affiliations:** 1 Eijkman Institute for Molecular Biology, Jakarta, Indonesia; 2 Department of Paediatrics, Dr. Cipto Mangunkusumo National Central Hospital, Faculty of Medicine, Universitas Indonesia, Jakarta, Indonesia; 3 Division of Vector-Borne Diseases, Centers for Disease Control and Prevention, Fort Collins, Colorado, United States of America; CDC, UNITED STATES

## Abstract

Although neurological manifestations associated with dengue viruses (DENV) infection have been reported, there is very limited information on the genetic characteristics of neurotropic DENV. Here we describe the isolation and complete genome analysis of DENV serotype 3 (DENV-3) from cerebrospinal fluid of an encephalitis paediatric patient in Jakarta, Indonesia. Next-generation sequencing was employed to deduce the complete genome of the neurotropic DENV-3 isolate. Based on complete genome analysis, two unique and nine uncommon amino acid changes in the protein coding region were observed in the virus. A phylogenetic tree and molecular clock analysis revealed that the neurotropic virus was a member of Sumatran-Javan clade of DENV-3 genotype I and shared a common ancestor with other isolates from Jakarta around 1998. This is the first report of neurotropic DENV-3 complete genome analysis, providing detailed information on the genetic characteristics of this virus.

## Author summary

Dengue viruses (DENV) are viruses that can cause asymptomatic infection to life-threatening haemorrhagic fever disease. Although DENV are not classically known to infect and invade central nervous system (CNS) in human, numerous cases of DENV infection in the CNS have been reported with limited information about the characteristics of the infecting virus. Here, we report the isolation and first complete genome analysis of DENV serotype 3 (DENV-3) from cerebrospinal fluid of a patient diagnosed with dengue encephalitis in Jakarta, Indonesia. By using next-generation sequencing strategy, we recovered the complete genome of the virus isolate and identified unique amino acid changes not found in any other recovered DENV-3 strains. The virus was determined to be closely related to isolates from Jakarta, Indonesia, which have been circulating for almost four decades.

## Introduction

Dengue viruses (DENV) are among the most important mosquito-borne viruses from the genus *Flavivirus*, family *Flaviviridae* and have been a major public health problem in many parts of the world, including Southeast Asia and the Americas [1]. In Indonesia, DENV has become a significant public health problem with a trend toward increasing numbers of outbreaks [2–5]. Infection with any of the four DENV serotypes can be asymptomatic or cause a spectrum of clinical symptoms from mild fever to a more severe, potentially life-threatening disease including dengue haemorrhagic fever and shock syndrome [6]. Although DENV is not a classical neurotropic virus, evidence of DENV neurotropism and neurological dengue have increased over the past five decades [7]. In laboratory-confirmed cases of DENV infection with admission to the hospital, the frequency of neurological involvement has ranged between 0.5% to 21%; while in patients admitted to the hospitals with encephalitis or suspected central nervous system (CNS) infection, DENV was identified in 4⎼47% of patients in endemic areas [7]. To address this expanding clinical manifestation of DENV infection, the latest WHO dengue guidelines published in 2011 have included CNS involvement in the definition of severe disease [8]. Nevertheless, the molecular and biological characteristics of neurotropic DENV strains is extremely limited despite its important role in deciphering the neuropathogenesis of dengue. Here, we report the isolation and complete genome analysis of DENV-3 from the CSF on a paediatric encephalitis patient in Jakarta, Indonesia.

## Materials and methods

### Ethics statement

Informed consent was obtained from the parents of the patient. The patient’s identity and personal information has been de-identified from the sample number, test results, or GenBank accession number. The study was approved by the Eijkman Institute Research Ethic Commission (Ethical Approval No. 66).

### Case description and DENV detection

In June 2016, a 10-year old boy was referred to Dr. Cipto Mangunkusumo National Central Hospital, Jakarta, Indonesia with the chief complaint of decreased consciousness on the second day of illness. He had history of high grade fever for two days accompanied with headache, nausea, vomiting, seizures and altered consciousness before admission. His laboratory examination showed hemoconcentration, thrombocytopenia, positive anti-DENV serum IgM and NS1 antigen, and normal blood chemistries. Lumbar puncture (LP) was performed on day two post illness onset to obtain CSF sample from the patient. There was no evidence of blood in the CSF indicating that the LP was not traumatic. The CSF was clear with 5 polymorphonuclear cells/μl, 2 mononuclear cells/μl, 60 mg/dl protein, 51 mg/dl glucose, and 121 mEq/l chloride. The patient was diagnosed with dengue encephalitis based on the neurological symptoms and detection of anti-DENV antibody and NS1 antigen in serum and was given supportive therapy. His consciousness improved on day 5 of illness after intensive supportive care and he was discharged after 12 days without any neurological sequelae. The CSF sample (designated as 201610225), was submitted to Eijkman Institute where it was analysed by using pan-alphavirus and pan-flavivirus RT-PCR targeting nSP4 and NS5 genes, respectively [9,10]. Pan-alphavirus RT-PCR was negative, while pan-flavivirus RT-PCR was positive. A flavivirus-specific 265-bp amplicon was sequenced from the sample and showed 99% nucleotide identity with DENV-3 in subsequent BLAST analysis.

Further examination of the CSF and serum samples confirmed the presence of DENV-3 RNA by using Simplexa Dengue Real-time RT-PCR Kit (Focus Diagnostics, Cypress, CA). The anti-dengue IgM and anti-dengue IgG (Panbio Dengue Duo IgM and IgG Capture ELISA, Alere, Brisbane, Australia) were absent from CSF although the serum sample was DENV IgM-positive/IgG-negative, suggesting a primary DENV infection. DENV NS1 antigen was detected in the serum and CSF samples by using rapid NS1 detection kit (Panbio Dengue Early Rapid). The DENV viral load was measured by using SYBR Green qRT-PCR as described previously [11] and was estimated to be 19,800 and 975,000 PFU equivalent/ml in the CSF and serum samples, respectively.

### Virus isolation in cell culture and indirect immunofluorescent assay (IFA)

Virus isolation was performed by using Vero cells (African green monkey kidney epithelial) as described previously [12]. Cell monolayers were inoculated with the CSF and serum samples and incubated for 1 hour at 37°C. Infected monolayers were subsequently maintained in MEM medium supplemented with 10% FBS, 500 U/ml penicillin, 500 μg/ml streptomycin, and 2 mM L-glutamine (Gibco, Carlsbad, CA). The presence of cytopathic effect (CPE) was investigated daily and DENV-3 RNA in culture supernatant was detected by pan-flavivirus RT-PCR and sequencing of the amplicon.

Virus isolation from CSF sample was also performed by using *Aedes albopictus* C6/36 cells. Cell monolayer was inoculated with CSF sample and incubated for 1 hour at 28°C and subsequently maintained in RPMI medium supplemented with 10% FBS, 500 U/ml penicillin, 500 μg/ml streptomycin, and 2 mM L-glutamine (Gibco). The presence of CPE was investigated daily and DENV-3 infection was confirmed by IFA staining with anti-DENV-3 monoclonal antibody. In the absence of CPE within 10 days, culture supernatant was blind passaged to new C6/36 cell monolayer. For IFA staining, infected C6/36 cells were spotted onto 12-well Teflon coated slides, air-dried and then fixed in cold 100% acetone for 10 min. Fixed cells were incubated with mouse anti-DENV-3 monoclonal antibody (clone 5D4) for 30 minutes at 37°C, followed by FITC-conjugated goat anti-mouse IgG (Sigma, St. Louis, MO, USA) for 30 minutes at 37°C. Cells were rinsed twice with PBS and counterstained with 0.05% Evans blue.

### DENV full-genome analyses

Next-generation sequencing (NGS) was employed to sequence the complete DENV-3 RNA genome from Vero cell culture supernatant by using Ovation RNA-Seq System V2 library preparation kit (NuGEN, San Carlos, CA) according to the manufacturer’s recommendations. The Ion PGM Hi‐Q OT2 Kit (Life Technologies, Carlsbad, CA) was used to prepare and enrich template-positive particles from the cDNA library. Enriched cDNA library was sequenced using the Ion PGM sequencer, Ion PGM Hi‐Q Sequencing Kit, and an Ion 316 Chip v2 BC.

Reads from the Ion PGM sequencer were dereplicated and filtered based on their quality and length with PRINSEQ lite version (prinseq-lite.pl) [13], with following settings: -derep 5 -max_len 250 -min_len 125 -min_qual_mean 30. These settings were used to remove reverse complement 5'/3' duplicates as well as to screen for reads with Q>30 and length range 125–250 bp. SPAdes assembler v.3.7.1 meta-mode [14] was used to assemble the filtered reads, with following settings:--iontorrent -k 21,33,55 --meta.

The extreme 5’ and 3’UTR of the genome were obtained by Sanger sequencing using DENV-3-specific primer sets as described previously [15]. The full length 10,707-nt sequence of DENV-3 strain 201610225 was annotated with Genome Annotator tools in Virus Pathogen Database and Analysis Resource (ViPR, www.ViPRbrc.org).

### DENV-3 sequence and phylogenetic analysis

The complete genome sequence of DENV-3 strain 201610225 was assembled and aligned with DENV-3 prototype strain H87 and all available DENV-3 complete genome sequences retrieved from the GenBank database with known host and country of isolation as of December 6, 2016 (n = 787) (S1 Table). Multiple sequence alignment was performed using MUSCLE v.3.8 [16] and trimmed to generate a dataset of 10,170-nucleotide (3,390 amino acid) encoding the complete DENV-3 open reading frame (ORF). The aligned sequences were used to analyse amino acid sequence variation visualized in MEGA v.7.0.21.

The 3’UTR RNA secondary structures of 201610225 and prototype strain H87 were predicted using the Mfold web server (http://unafold.rna.albany.edu/?q=mfold) [17]. Mfold predictions were constrained to preserve consistency of RNA structures with bioinformatics and biochemical predictions as described previously [18]. The optimal energy folding patterns from the Mfold predictions were visualized with VARNA [19].

To identify representative and closely related reference sequences for deep phylogenetic analysis, a maximum-likelihood phylogenetic tree was constructed from the initial alignment by using FastTree v.2.1. The evolutionary history and the time to most common ancestor (TMRCA) of strain 201610225 was analysed using Bayesian Markov chain Monte Carlo (MCMC) method as implemented in BEAST v.1.7.4 [20]. The evolution model was evaluated using jModelTest v.2.2.7 [21] and the generalised time reversible (GTR) model with invariant and gamma sites (GTR+I+G) was used, as recommended by Akaike (AIC) and Bayesian information criteria (BIC) from jModelTest output. BEAST analysis was subsequently performed with following parameters: GTR+I+G, lognormal relaxed clock, uniform clock rate, coalescent Bayesian skyride tree prior, and 50 million MCMC repetitions (sampling every 1,000 trees) [22]. The collection of trees from this process was annotated using TreeAnnotator v.1.8.2 to get the most credible tree. FigTree v1.2.2 was used to view and evaluate the most credible tree. Similarly, for further grouping of 201610225 relative to other recent Indonesian DENV-3 strains, 112 available DENV-3 envelope gene sequences (1,479 nt) were analysed including prototype reference strains for each DENV-3 genotype as well as recent Indonesian and Southeast Asian strains.

Amino acid changes mapping onto existing homologous structural data from Protein Data Bank (PDB) was done by using FeatureMap3D (http://www.cbs.dtu.dk/services/) [23]. The multiple sequence alignment dataset was also used to check for the presence of recombination in our isolate by using the Recombination Detection Program version 4 (RDP4) which used seven different recombination detection methods [24]. A sequence was considered as a potential recombinant only if it was detected as significant (with *p*-value cutoff of 0.00001) by at least two methods.

### Accession number

The complete genome sequence of DENV-3 isolate 201610225 is available in GenBank under accession number KY863456.

## Results

**Fig 1:**
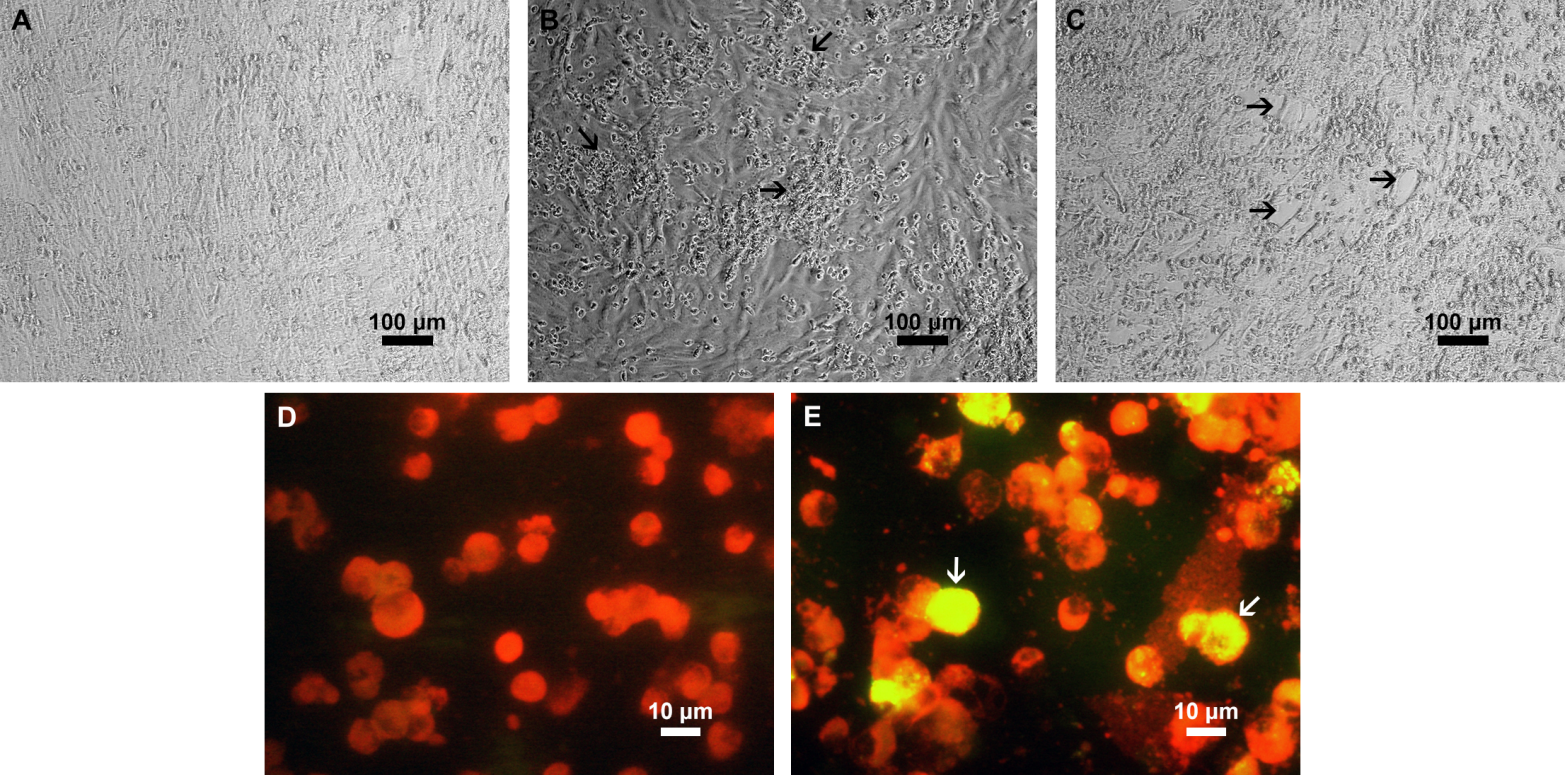
Isolation of DENV-3 from CSF and serum samples of an encephalitis patient in Vero and C6/36 mosquito cell cultures. Phase contrast light microscopy of uninfected monolayer Vero cells (A); infected Vero cells at day 5 after inoculation with CSF sample (B); and infected Vero cells at day 10 after inoculation with serum sample (C). Indirect immunofluorescent assay (IFA) staining of uninfected C6/36 mosquito cells (D); infected C6/36 mosquito cells at third passage of inoculation with CSF sample (E). For IFA staining, cells were stained with mouse anti-DENV-3 monoclonal antibody (clone 5D4), followed by FITC-conjugated goat anti-mouse IgG. Evans blue was used as a counterstain (red). The arrows in B and C point to cytopathic effect (CPE), while the arrows in E point to DENV-infected cells.

Neurotropic DENV-3 strain was isolated and identified from CSF sample of a paediatric encephalitis patient in Jakarta, Indonesia. Inoculation of CSF sample from the patient into Vero cells caused apparent CPE after day 5 (Fig 1B), while inoculation of the serum sample caused distinguishable CPE after day 10 (Fig 1C). No changes were observed in the uninfected Vero cells (Fig 1A). Infection of the Vero cell was confirmed by detection of DENV-3 RNA in the culture supernatant by pan-flavivirus RT-PCR followed by sequencing of the amplicon. Furthermore, DENV-3 infection at third passage in C6/36 cells inoculated with CSF sample was confirmed by detection of intracellular DENV-3 antigen using DENV-3-specific IFA staining (Fig 1E). The presence of DENV-3 antigen was not observed in uninfected C6/36 cells (Fig 1D).

By using NGS strategy, 94% of the complete genome was determined from the virus isolate recovered from Vero cell culture supernatant, while the remaining 5’ and 3’UTR were sequenced by classical Sanger sequencing. The extreme terminal 5’ and 3’UTR were not confirmed independently (i.e. using RACE method) and assumed to be of the same length as the prototype DENV-3 strain H87. The complete genome of isolate 201610225 consists of 94 nucleotides of 5’UTR, 440 nucleotides of 3’UTR, and a 10,173-nucleotide ORF located from 95 to 10267.

Compared with the prototype DENV-3 strain H87, 46 amino acid changes were found within the genome of 201610225 (Table 1). Among these, 14 and 20 amino acid changes were shared with the historical DENV-3 strain collected in Sleman in 1978 (Sleman/78) and Jakarta in 1988 (den3_88), respectively. Two novel amino acid changes, R3259K and F3369Y, located in the NS5 protein coding sequence were not observed in any other complete DENV-3 genome. However, when these two changes were mapped to the 3D structure of the NS5 proteins, we found that R3259K and F3369Y are not predicted to significantly affect the physicochemical property of the NS5 protein although the R3259K change is located in the protein bend structure. Moreover, while the remaining 44 amino acid changes were also observed in other DENV-3 strains in our complete genome data set, nine amino acid changes were only found in relatively few other DENV-3 strains (less than 1% occurrence), including G76R (found in 4 others), A624V (found in 2 others), I896V (found in 3 others), R1176K (found in 2 others), H1180Q (found in 1 others), K1658R (found in 6 others), Q1940R (found in 1 other), D2764N (found in 2 others), and Q3052R (found in 7 others).

**Table 1:** Amino acid substitutions between 201610225, DENV-3 prototype strain (H87), and DENV-3 Indonesian historical strain (Sleman/78 and den3_88) genome sequence.

DENV-3 gene	Gene position ^a^	Polyprotein position ^b^	Amino acid residue				No. of other 784 DENV-3 genome sequences that share 201610225 aa changes (%)
			H87 (KU050695)	Sleman/78 (AY648961)	den3_88 (AY858038)	201610225	
C	65	65	V	V	I	I	35 (4.46)
	76	76	G	G	G	R ^c^	4 (0.51)
	82	82	K	R	R	R	59 (7.53)
	97	97	K	R	R	R	60 (7.65)
pr	55	169	H	L	L	L	209 (26.66)
M	16	221	T	T	T	A	27 (3.44)
	37	242	L	F	F	F	60 (7.65)
E	68	348	I	V	V	V	61 (7.78)
	124	404	S	S	S	L	30 (3.83)
	169	449	A	V	V	V	213 (27.17)
	231	511	R	K	K	K	66 (8.42)
	301	581	L	L	L	S	25 (3.19)
	303	583	T	A	A	A	69 (8.80)
	344	624	A	A	A	V ^c^	2 (0.26)
	377	657	V	V	V	I	25 (3.19)
	479	759	A	V	A	V	225 (28.70)
NS1	96	869	V	V	V	I	15 (1.91)
	123	896	I	I	I	V ^c^	3 (0.38)
	145	918	S	S	S	N	38 (4.85)
	174	947	V	M	M	M	48 (6.12)
	256	1029	H	Y	Y	Y	321 (40.94)
NS2A	51	1176	R	R	R	K ^c^	2 (0.26)
	55	1180	H	H	R	Q ^c^	1 (0.13)
	153	1278	T	M	M	M	50 (6.38)
NS3	60	1533	H	H	H	Y	524 (66.84)
	185	1658	K	K	K	R ^c^	6 (0.77)
	255	1728	R	R	K	K	35 (4.46)
	350	1823	E	E	D	D	25 (3.19)
	467	1940	Q	Q	Q	R ^c^	1 (0.13)
NS4A	78	2170	I	I	I	V	21 (2.68)
	89	2181	I	A	V	V	60 (7.65)
	100	2192	V	V	V	I	596 (76.02)
NS4B	190	2432	L	F	F	F	60 (7.65)
NS5	188	2678	T	T	T	A	46 (5.87)
	246	2736	H	H	H	Y	29 (3.70)
	274	2764	D	D	D	N ^c^	2 (0.26)
	281	2771	K	R	R	R	63 (8.04)
	336	2826	M	M	T	T	36 (4.59)
	364	2854	R	R	R	K	26 (3.32)
	422	2912	R	R	R	K	572 (72.96)
	562	3052	Q	Q	Q	R ^c^	7 (0.89)
	631	3121	A	A	A	T	38 (4.85)
	749	3239	R	R	K	K	327 (41.71)
	763	3253	T	T	A	A	61 (7.78)
	769	3259	R	R	R	K ^d^	0 (0.00)
	879	3369	F	F	F	Y ^d^	0 (0.00)

^a^Amino acid numbering is given from the start of each gene of DENV-3 prototype strain H87.

^b^Amino acid numbering is given relative to the complete coding region of DENV-3 prototype strain H87.

^c^Denotes amino acid changes which were found in relatively few other DENV-3 strains (less than 1% occurrence)

^d^Denotes amino acid changes which are unique to isolate 201610225

**Fig 2:**
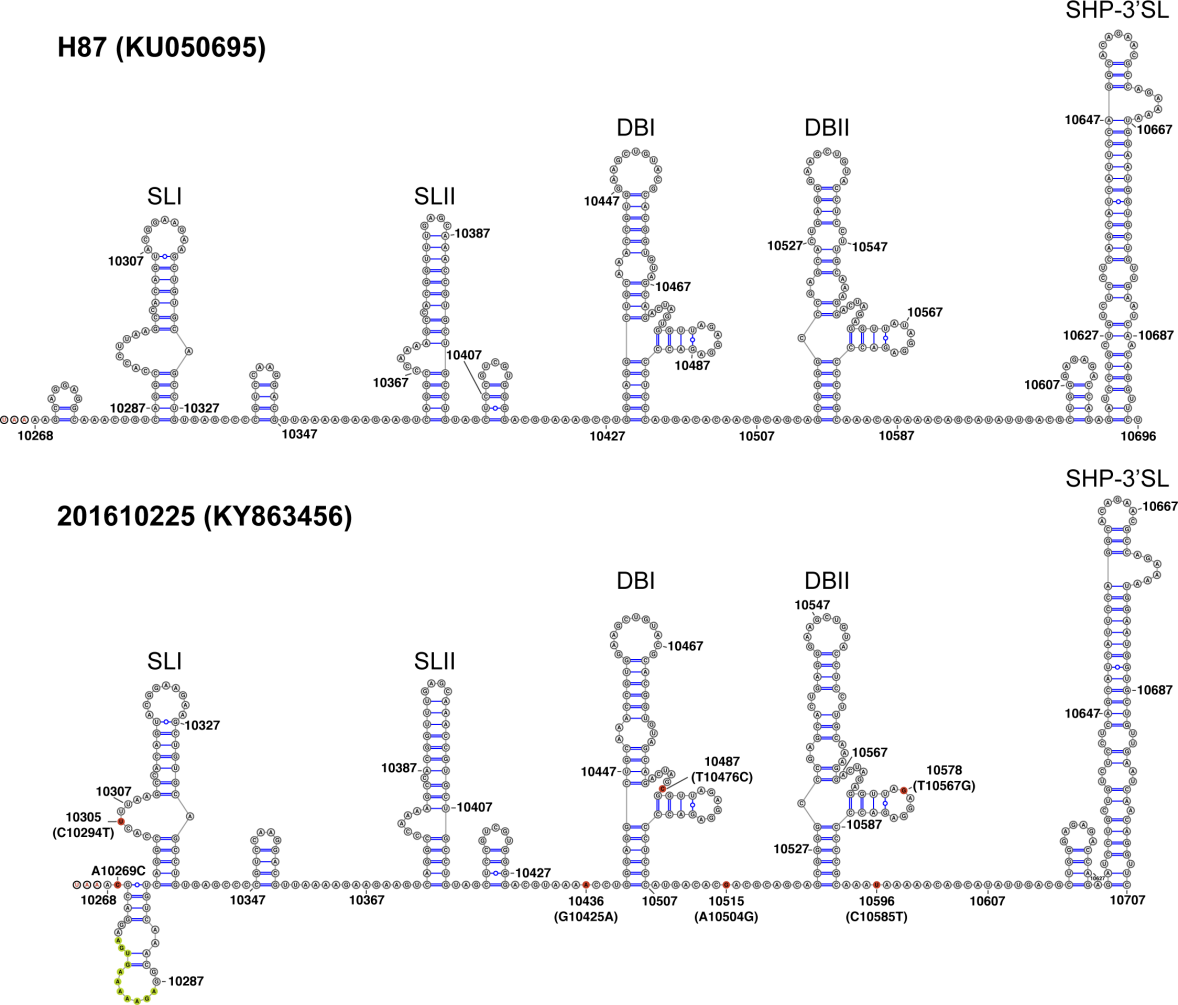
Mfold RNA structure prediction for the complete 3’UTR of DENV-3 prototype strain H87 (KU050695) and the neurotropic DENV-3 201610225 (KY863456). Stop codon (UAA) is indicated with red font in both RNA structure just upstream nucleotide 10268. Nucleotide insertion and changes in isolate 201610225 are indicated with yellow and red circles, respectively. The positions of the nucleotide changes are indicated according to 201610225 nucleotide numbering with H87 nucleotide numbering in parentheses. The two stem-loops (SLI and SLII), two dumbbells (DBI and DBII), and small hairpin 3’ stem-loop (SHP-3’SL) structures are shown.

In addition to amino acid changes within the ORF, we also examined the nucleotide changes that occurred in the 5’UTR and 3’UTR of the neurotropic DENV-3 genome. Compared with the prototype DENV-3 strain H87, a G to A change at position 62 was observed; this change was also seen in other DENV-3 strains in our data set. The G62A nucleotide change was found to be associated with DENV-3 genotype I specifically [25]. No other nucleotide changes were found in the 5’UTR of our neurotropic DENV-3 genome. The 3’UTR of 201610225 genome has an insertion of an 11-nucleotide sequence (AGTGAAAAAGA) after position 10275 and seven nucleotide changes including A10269C, C10294T, G10425A, T10476C, A10504G, T10567G, and C10585T when compared with the prototype DENV-3 strain H87. The RNA structure of complete 3’UTR 201610225 was predicted and compared with the strain H87 (Fig 2). The nucleotide changes present in our isolate were not predicted to alter the two stem-loop (SLI and SLII), the conserved duplicated dumbbell structures (DBI and DBII), or the essential terminal structure small hairpin 3’ stem-loop (SHP-3’SL) which are important for viral RNA synthesis [18]. However, the 11-nucleotide insertion observed in our isolate was predicted to form bigger stem-loop structure downstream of the stop codon compared with the strain H87. Furthermore, when we compared these nucleotide insertion and changes to all other DENV-3 3’UTR sequences, we found that none were unique to isolate 201610225.

**Fig 3:**
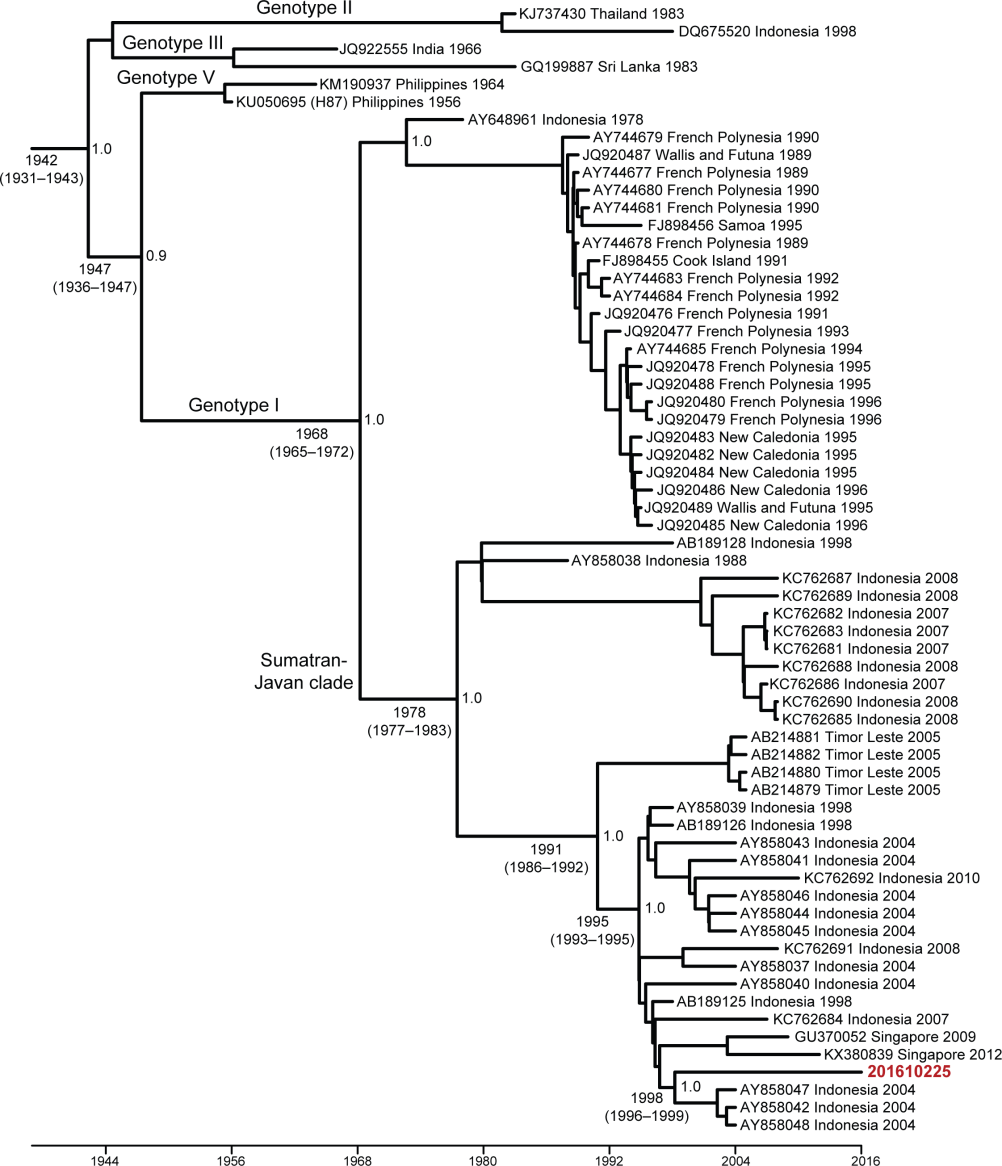
Maximum clade credibility (MCC) tree of 64 complete coding sequences of DENV-3 and the neurotropic DENV-3 isolate 201610225. Horizontal branches are drawn to scale of estimated year of divergence with tip times reflecting sampling date (year). The coalescent (i.e. divergence) times of key nodes, 95% HPD values, as well their posterior probability are shown. Strains are labeled as follows: GenBank accession number/country/year. Isolate 201610225 presented here is in bold red font. GenBank accession number for the complete genome sequence 201610225 is KY863456.

The complete ORF of DENV-3 phylogenetic tree generated using the Bayesian MCMC method showed that 201610225 belongs to genotype I based on Lanciotti *et al* and Wittke *et al* classification [26,27] and is closely related to isolate TB16, KJ30i, and TB55i (GenBank acc. no. AY858047, AY858042, and AY858048, respectively) sampled in Jakarta, Indonesia in 2004 (Fig 3). This phylogenetic history was supported by high posterior probability values (1.0). Molecular clock analysis suggested that these strains shared a common ancestor dated from approximately 1998 (95% HPD: 1996–1999). Furthermore, our phylogenetic tree also indicates that 201610225 was part of Sumatran-Javan clade which includes DENV-3 genotype I strains from Sumatra, Jawa, and Sulawesi islands in Indonesia as well as strains from Timor Leste (formerly East Timor) described previously [28]. The time to the most recent common ancestor (TMRCA) of this Sumatran-Javan clade was approximately 1978 (95% HPD: 1977–1983) supported by high posterior probability values (1.0). Similar to the complete ORF phylogenetic tree, MCMC phylogenetic tree based on the envelope gene with additional recent DENV-3 Indonesian and Southeast Asian reference strains suggested that strain 201610225 is closely related to the same Jakarta strains mentioned above as well as to strains from Bandung (GenBank acc. no. AY265857) and Semarang (GenBank acc. no. KC589013 and KC589012), Jawa island, Indonesia (S1 Fig). The TMRCA of this clade based on the envelope region is estimated to be 1997 (95% HPD: 1996–1998) with high posterior probability values (1.0). The envelope phylogenetic tree also confirms the classification of our isolate as DENV-3 genotype I. Additionally, screening for recombination by RDP4 analysis found no evidence of recombination in 201610225 genome.

## Discussion

Over the last five decades, numerous studies have reported DENV-associated neurological manifestations in prospective and retrospective as well as in outbreak and case report study settings [7]. Despite the recent increase in the number of cases of neurological dengue, the pathogenesis of neurotropism is still controversial and the neurotropic virus are not well characterized, although some studies suggest a direct virus-induced destruction of neurons [29–31]. The encephalitis case presented here is similar to cases described in some of the earliest reports of DENV-associated encephalitis/encephalopathy in Indonesia [32–35]. However, assays to detect virus or antibody were either not performed or not successful in these studies. Our report describes the isolation and complete genome analysis of DENV-3 from CSF of an encephalitis patient in Jakarta, Indonesia. Although a number of studies have reported the presence of DENV RNA and infectious virus in the CSF of encephalitis patients, there are only few published studies detailing the findings on the genetic characteristics of the neurotropic DENV strains, including one DENV-4 complete genome sequence, one DENV-3 partial capsid-prM sequence, one DENV-3 envelope, and two DENV-2 partial envelope-NS1 sequences isolated directly from CSF of encephalitis patients [36–39]. To our knowledge, this is the first complete genome analysis of DENV-3 isolated from CSF in a patient with neurological manifestations, underscoring the need for more molecular studies to better characterize neurotropic DENV strains.

The identification of serotype DENV-3 in an encephalitis case is not unexpected, as DENV-3, together with DENV-2, appeared to be more frequently associated with encephalitis and other neurological complications compared with other serotypes [29,37–43,35]. Although all DENV serotypes are endemic in Indonesia and cycling occurs between DENV serotypes, DENV-3 has been historically reported as the predominant circulating serotype since 1975 [44]. A meta-analysis study encompassing 15,741 cases of dengue from 31 reports of primary infections found that these primary infections with DENV-3 are associated with severe cases under WHO new classification [45], thus emphasizing the need for appropriate clinical consideration for DENV-3 cases.

It is also noteworthy that the patient in this report experienced primary DENV infection as evidenced by the absence of anti-DENV IgG both in serum and CSF. Antibody-dependent enhancement (ADE) in secondary DENV infections has been proposed to increase viral replication leading to over activation of immune response [46]. However, the neurological complications seen in this patient might be the consequences of direct viral invasion in the CNS, rather than non-specific immune complications of DENV infection. The finding of DENV in CSF also supports a more direct cause of viral invasion as with earlier studies [31,36,37]. The presence of relatively high viral load in the serum compared to CSF is also striking in contrast to other flaviviruses such as Japanese encephalitis virus and West Nile virus infections, where viremia is undetectable by the time they invade the CNS [47–49]. Nevertheless, dengue encephalitis could be due to host genetic or immunologic differences rather than viral mutations although evidence to support this possibility is scarce. Many studies have shown that dengue disease severity can be influenced by host genetic variation and immune status [reviewed in 6]. However, these studies did not specifically compare dengue encephalitis and non-encephalitis patients and further data from dengue encephalitis cases is still needed.

In this study, two unique and nine uncommon amino acid changes were shown to be present in isolate 201610225. The two unique amino acid changes in NS5 region (R3259K and F3369Y) have never been reported before, while the nine uncommon amino acid changes including G76R (capsid); A624V (envelope); I896V (NS1); R1176K and H1180Q (NS2A); K1658R and Q1940R (NS3); D2764N and Q3052R (NS5) were found in a few other strains with complete genomes listed in GenBank. The putative amino acid changes associated with DENV-3 neurovirulence in mouse [50], including A18S, A54E, F277S, E401K, and T403I were not found in isolate 201610225. Similarly, the amino acid change in the envelope protein (E406K) which has been identified as a neurovirulence determinant of DENV-2 and DENV-3 [51,52] was not found in isolate 201610225. Furthermore, no amino acid changes are shared between isolate 201610225 and partial genome sequences of DENV-3 strains isolated from the CSF of encephalitis patients described earlier (DENV-3 partial capsid-prM and envelope sequence; GenBank acc. no. KM024988 and KP893717, respectively). Interestingly however, amino acid changes F3369Y and K1658R found in our neurotropic DENV-3 were also found in corresponding position of neurotropic DENV-4 (GenBank acc. no. JX024757) [36]. The amino acids F3369Y and K1658R shared similar properties; F and Y are aromatic hydrophobic amino acids with Y being more polar, while K and R are positively-charged polar amino acids with K being more hydrophobic. Whether these amino acid changes represent genetic determinant of DENV neurovirulence is unknown. Genetic characterization of additional neurotropic DENVs will allow further insights into the role of these unique amino acid changes found in isolate 201610225. In addition, further studies are warranted to study the virulence of this isolate in animal models and its tropism for neural cells *in vitro*.

Complete genome phylogenetic analysis placed the 201610225 strain in the DENV-3 genotype I group, reported to be circulating in Indonesia for almost four decades. It has been suggested by Ong *et al* [28] that the strains from this particular clade are likely to cause another dengue epidemic if they remain in circulation. Not surprisingly, within the Sumatran-Javan clade of DENV-3 genotype I, isolate 201610225 was found to be more closely related to isolates from Jakarta and Singapore than to those from Makassar, a city on eastern Indonesia’s Sulawesi island. Together, this result suggests the endemicity of Sumatran-Javan clade of DENV-3 with sustained transmission in Indonesia.

In summary, we described here the isolation and the first complete genome analysis of DENV-3 from the CSF of an encephalitis patient. Our findings complement those of others in suggesting the greater pathogenic potential of DENV-3 to cause neurological complications. Furthermore, DENV should be included in the laboratory diagnostic algorithm for encephalitis and other CNS infections especially in dengue endemic areas, despite the challenges in laboratory confirmation as isolation of virus or antibody from CSF is often difficult.

## Supporting information

S1 Fig Maximum clade credibility (MCC) tree of 112 envelope gene sequences of DENV-3 and the neurotropic DENV-3 isolate 201610225. Horizontal branches are drawn to scale of estimated year of divergence with tip times reflecting sampling date (year). The coalescent (i.e. divergence) times of key nodes, 95% HPD values, as well their posterior probability are shown. Strains are labeled as follows: GenBank accession number/country/year. Isolate 201610225 presented here is in bold red font.
(PDF)

S1 Table List of all available DENV-3 complete genome sequences retrieved from the GenBank database with known host and country of isolation as of December 6, 2016. (XLSX)
