# Supplementary figures and images for: Isolation and complete genome analysis of neurotropic dengue virus serotype 3 from the cerebrospinal fluid of an encephalitis patient
Source: PLoS Negl Trop Dis. 2018 Jan 12;12(1):e0006198. doi: 10.1371/journal.pntd.0006198 (PMC5809095; doi:10.1371/journal.pntd.0006198)

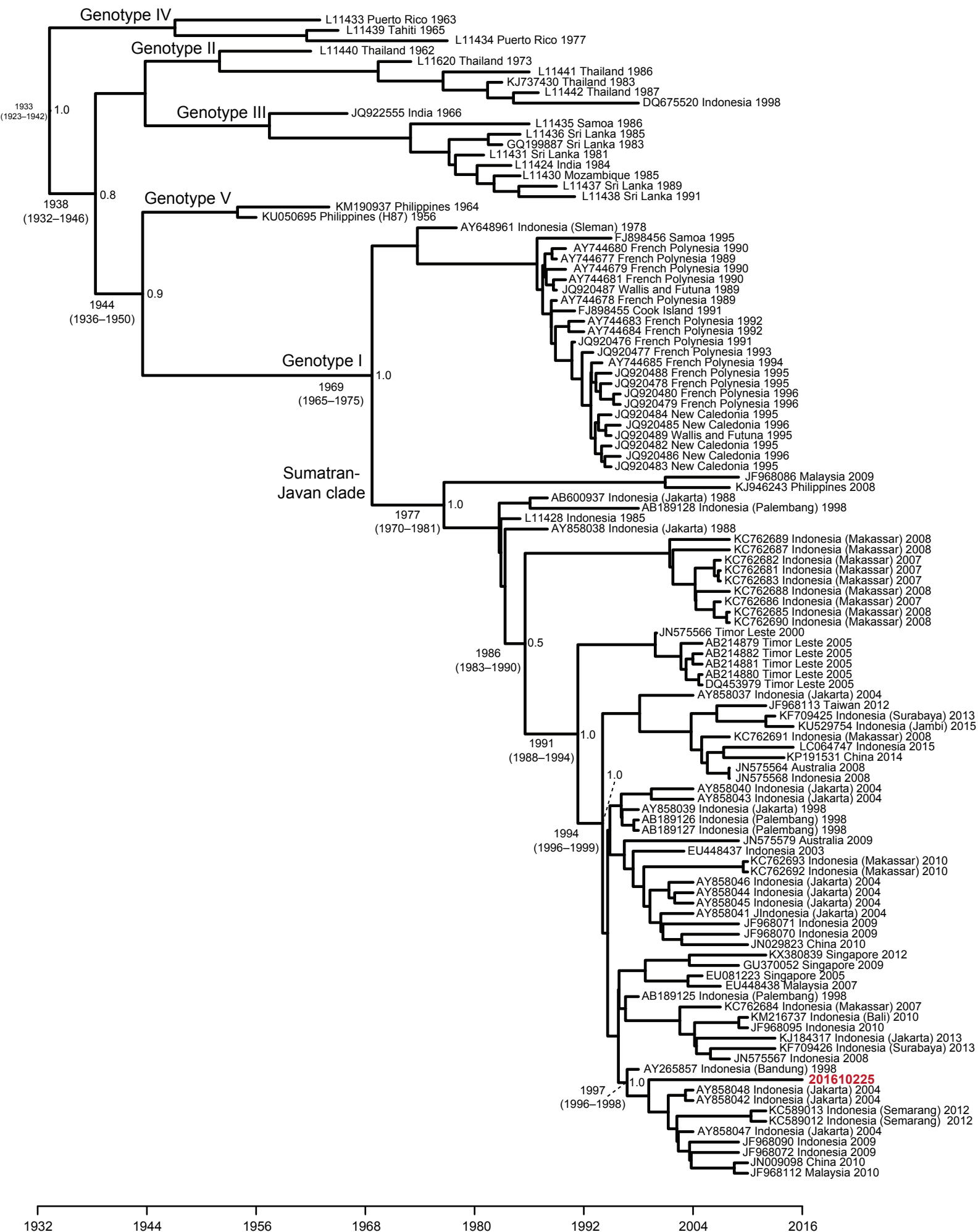

Supplement: S1 Fig — Horizontal branches are drawn to scale of estimated year of divergence with tip times reflecting sampling date (year). The coalescent (i.e. divergence) times of key nodes, 95% HPD values, as well their posterior probability are shown. Strains are labeled as follows: GenBank accession number/country/year. Isolate 201610225 presented here is in bold red font. (PDF) [file pntd.0006198.s001.pdf]
